# Molecular Analysis of Prognosis and Immune Pathways of Pancreatic Cancer Based on TNF Family Members

**DOI:** 10.1155/2021/2676996

**Published:** 2021-09-30

**Authors:** Zemin Zhu, Caixi Tang, Tao Xu, Zhijian Zhao

**Affiliations:** Department of Hepatobiliary and Pancreatic Surgery, Zhuzhou Central Hospital, Zhuzhou, China

## Abstract

**Background:**

Tumor necrosis factor (TNF) family members play a vital role in anticancer therapy. This study aimed to screen the critical markers for the prognostic analysis of pancreatic adenocarcinoma (PAAD) by analyzing the clustering patterns of TNF family members in PAAD.

**Methods:**

In this study, the NMF clustering method was adopted to cluster samples from The Cancer Genome Atlas (TCGA) to acquire the clustering pattern of the TNF family in PAAD. Differential gene analysis was performed according to TNF family gene clusters. The support vector machine (SVM) method was conducted for further gene screening, and the risk score model of the screened genes was constructed by Lasso. The single sample gene set enrichment analysis (ssGSEA) method was adopted for immunoenrichment analysis and tumor immune cycle analysis. Genes associated with risk scores were analyzed by Gene Ontology (GO) and Kyoto Encyclopedia of Genes and Genomes (KEGG) enrichment analysis.

**Results:**

We clustered PAAD into two groups based on TNF family genes. Nineteen TNF family genes were significantly associated with the clinical characteristics of PAAD patients. The risk score formula was composed of RHOD, UBE2C, KLHDC7b, MSLN, ADAM8, NME3, GNG2, and MCOLN3. GSE57495 and GSE62452 datasets verified that patients in the high-risk group had a worse prognosis than those in the low-risk group. The risk score-related genes analyzed by GO and KEGG were mainly involved in the modulation of chemical synaptic transmission and synaptic vesicle cycle pathway. There were significant differences in the expression of 15 immune cells between the high-risk group and the low-risk group. The risk score was positively correlated with HCK, interferon, MHC-I, and STAT1. The expression of genes relevant to chemokine, immunostimulator, MHC, and receptor was strongly associated with the risk score.

**Conclusion:**

The risk score model based on the TNF family can predict the prognosis and immune status of PAAD patients. Further research is needed to verify the clinical prognostic value of risk scores.

## 1. Introduction

Pancreatic adenocarcinoma (PAAD) has a 5-year survival rate of less than 10%, which is one of the most aggressive malignant tumors with the worst prognosis [1, 2]. About 90% of PAADs are ductal adenocarcinomas originating from the glandular epithelium. In recent years, the morbidity and mortality of the disease have increased significantly. Studies have predicted that PAAD may become the second leading cause of cancer-related death after 2030 [3]. Low immunophenotype and low tumor sensitivity to cytotoxic drugs are the primary causes for the low survival rate in PAAD patients [4]. The cytotoxic chemotherapy for PAAD is primarily limited to the absence of molecular markers to predict the efficacy of chemotherapy [5]. The lack of a specific tumor prognostic model is a serious challenge in the treatment of PAAD. Therefore, more efforts are urgently needed to understand the specific prognostic markers and immune function of PAAD.

TNF and TNF receptor (TNFR) superfamily (TNFSF/TNFRSF) consist of nineteen ligands and twenty-nine receptors [6]. TNF family members are expressed naturally by the immune system and kill tumor activity [7]. In addition, tumor necrosis factor- (TNF-) related ligands have high expectations in anticancer therapy due to their induction of apoptosis [8]. For example, tumor necrosis factor-related apoptosis-inducing ligand (TRAIL), a member of the TNF family, has been shown to selectively induce apoptosis in cancer cells by binding or trimerizing its functional receptors [9]. In PAAD tissues, tumor-infiltrated immature M0 macrophages exhibit antitumor activity by secreting TNF-*α* [10]. Although a large body of evidence has shown that TNF family members play an essential role in many cancers, including PAAD, the role of TNF family members in PAAD is still not systematically understood.

In recent years, bioinformatics analysis of biodata information obtained from the public databases has played a very positive role in better understanding and treatment of several diseases, including cancer. Using algorithms such as ssGSEA, the expression level of markers can reflect the infiltration of specific cell types in tumor tissue. Through the complete follow-up data of multiple cohorts, the relevance between the relative infiltration level of specific cell types and the survival rate of patients can be determined [11]. For instance, based on the epigenetic properties of immunomodulatory cytokine genes, methylation of these genes has been found to be related to overall survival (OS), disease-specific survival, and disease progression in PAAD patients [12]. Yao et al. discovered differential splicing of AS events between PAAD and normal tissues and successfully constructed a prognostic model to predict the prognosis of PAAD patients using survival-related splicing factors [13]. More recently, Zhang et al. constructed a prognostic model based on the TNF family to predict the prognosis and immune status of lung adenocarcinoma (LUAD) patients [14]. In addition, it has been found that ubiquitin-specific protease 4 (USP4) plays a tumor-promoting role in PAAD and can be used as a prognostic indicator and therapeutic target in patients with PAAD resection [15]. However, no details of expression of TNF family members in PAAD and their clinical significance have been reported.

This study was a systematic study of expression patterns of TNF family members and their clinical significance in PAAD. We aimed to establish a prognostic model for PAAD based on the TNF family by in-depth analysis of relevant data from the TCGA and gene expression integration (GEO) databases. We hope that the prognostic risk score of this study will contribute to the prognosis of PAAD and the formulation of phase immunotherapy strategies.

## 2. Materials and Methods

### 2.1. Datasets and Preprocessing

The TCGA pancreatic adenocarcinoma dataset (TCGA-PAAD) was downloaded from the TCGA website, and 178 samples were included in the analysis. GSE57495 and GSE62452 datasets were downloaded from GEO (https://www.ncbi.nlm.nih.gov/geo/). Affymetrix generated raw data from the microarray dataset. The RMA algorithm in the Affy package was then applied to perform quantile normalization and background correction for Affymetrix raw data. GSE57495 included 63 samples, and GSE62452 included 66 samples.

### 2.2. Clustering Based on the TNF Family

TNF family was obtained as per the previous paper [14]. TCGA-PAAD was clustered by the NMF clustering method, and the clustering pattern based on the TNF family was obtained.

### 2.3. Establishment of the Risk Score Model

According to the TNF family gene cluster, the differential gene analysis was performed for the two clusters. The differential gene of standard was defined as |logFC| > log2(1.5) with *P* < 0.05. The independent prognostic significance of TNF family members was assessed by univariate Cox regression analysis (*P* < 0.05). HR > 1 was considered a prognostic risk gene, while HR < 1 was considered a protective prognostic gene. Univariate analysis was carried out for differential genes, and then SVM was used for further screening. The selected genes were modeled using Lasso, and the risk score was the sum of gene expression value ^*∗*^ Lasso coefficient.

### 2.4. Immunoinfiltration Analysis

The ssGSEA method was used for immunoinfiltration analysis. The expression levels of twenty-eight types of cells were mainly analyzed, including immature dendritic cell, immature B cell, activated B cell, activated CD4 T cell, activated CD8 T cell, macrophage, mast cell, MDSC, memory B cell, monocyte, activated dendritic cell, CD56bright natural killer cell, CD56dim natural killer cell, central memory CD4 T cell, central memory CD8 T cell, effector memory CD4 T cell, effector memory CD8 T cell, natural killer cell, natural killer T cell, neutrophil, plasmacytoid dendritic cell, regulatory T cell, follicular T-helper cell, type 1 T-helper cell, type 17 T-helper cell, type 2 T-helper cell, eosinophil, and gamma delta T cell [16]. Tumor immune-cycle analysis mainly analyzed seven steps of immune activity [17].

### 2.5. Pathway Analysis

Correlation analysis was performed on risk score and all genes, and the correlation standard was defined as |cor| > 0.6. Related genes were analyzed for functional enrichment, mainly by GO and KEGG analysis.

### 2.6. Statistical Analysis

The R package SurvMiner was used to draw all survival curves. The normality of variables was checked by the Shapiro–Wilk normality test. The unpaired Student's *t*-test was used to compare the differences between the two groups for variables that conform to the normal distribution. The nonnormally distributed variables were analyzed by the Wilcoxon test. The Kaplan–Meier method was used to generate and visualize subgroup survival curves. The logarithmic rank test was used to determine the statistical significance of the differences in each dataset. All heat maps were generated based on PHEATMAP. All statistical analyses were performed in R (https://www.r-project.org/, version 3.5.1). All the tests were two-sided, and *P* values < 0.05 were considered statistically significant.

## 3. Results

### 3.1. TNF Family Gene Clustering

**Figure 1:**
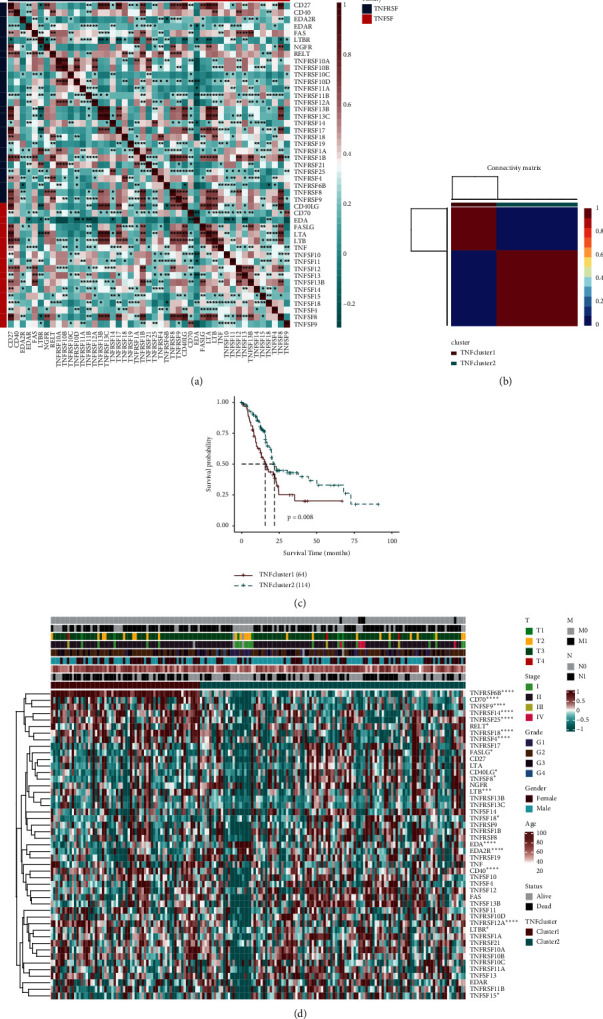
TNF family genes clustering in PAAD. (a) Correlation analysis of TNF family genes in the TCGA-PAAD dataset. Red is considered positive correlation, and green is considered negative correlation. (b) According to TNF family genes, PAAD was clustered into two groups, TNF-Cluster1 (red) and TNF-Cluster2 (blue). (c) The survival probability analysis of the two TNF clusters. (d) Gene expression maps of the TNF family in two TNF clusters. ^*∗∗*^*P* < 0.01. A prognostic model of PAAD based on TNF family genes was established.

First, we conducted a centralized association analysis of TNF family genes including 29 TNFRSF members and 18 TNFSF members (Figure 1(a)). According to TNF family genes, PAAD was clustered into TNF-Cluster1 and TNF-Cluster2 (Figure 1(b)). Based on the survival probability of the two TNF clusters, we found that the survival probability of TNF-Cluster1 (*n* = 64) was significantly lower than that of TNF-Cluster2 (*n* = 114) (*P* = 0.008) (Figure 1(c)). The expression of TNF family genes in the two TNF clusters is shown in Figure 1(d), among which 19 TNF family genes (TNFRSF6B, CD70, TNFSF9, TNFRSF14, TNFRSF25, RELT, TNFRSF18, TNFRSF4, FASLG, CD40LG, TNFSF8, LTB, TNFSF18, EDA, EDA2R, CD40, TNFRSF12 A, LTBR, and TNFSF15) were significantly correlated with the clinical characteristics (M, N, T, stage, grade, gender, and age) of PAAD patients. Among 47 TNF family genes, we found 14 dangerous prognostic genes and 1 protective prognostic gene (*p* ＜ 0.05, Figure S3).

**Figure 2:**
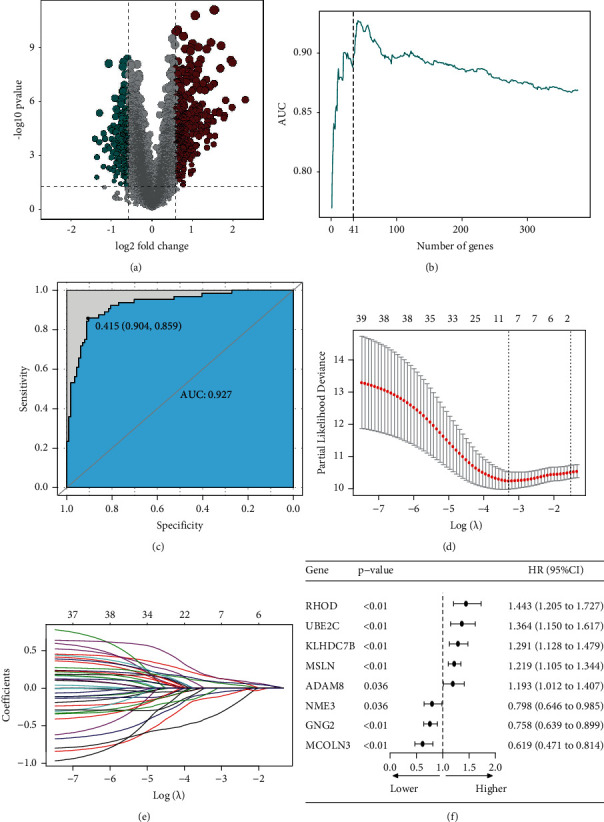
The risk score model of 8 TNF family genes. (a) A volcanic plot of TNF-Cluster1 and TNF-Cluster2 differential genes. (b) The number of genes obtained by the SVM (support vector machine) method after single-factor screening. (c) ROC curve of the clustering type was deduced by the SVM model. (d–f) The risk score model of 8 genes was obtained after Lasso analysis.

**Figure 3:**
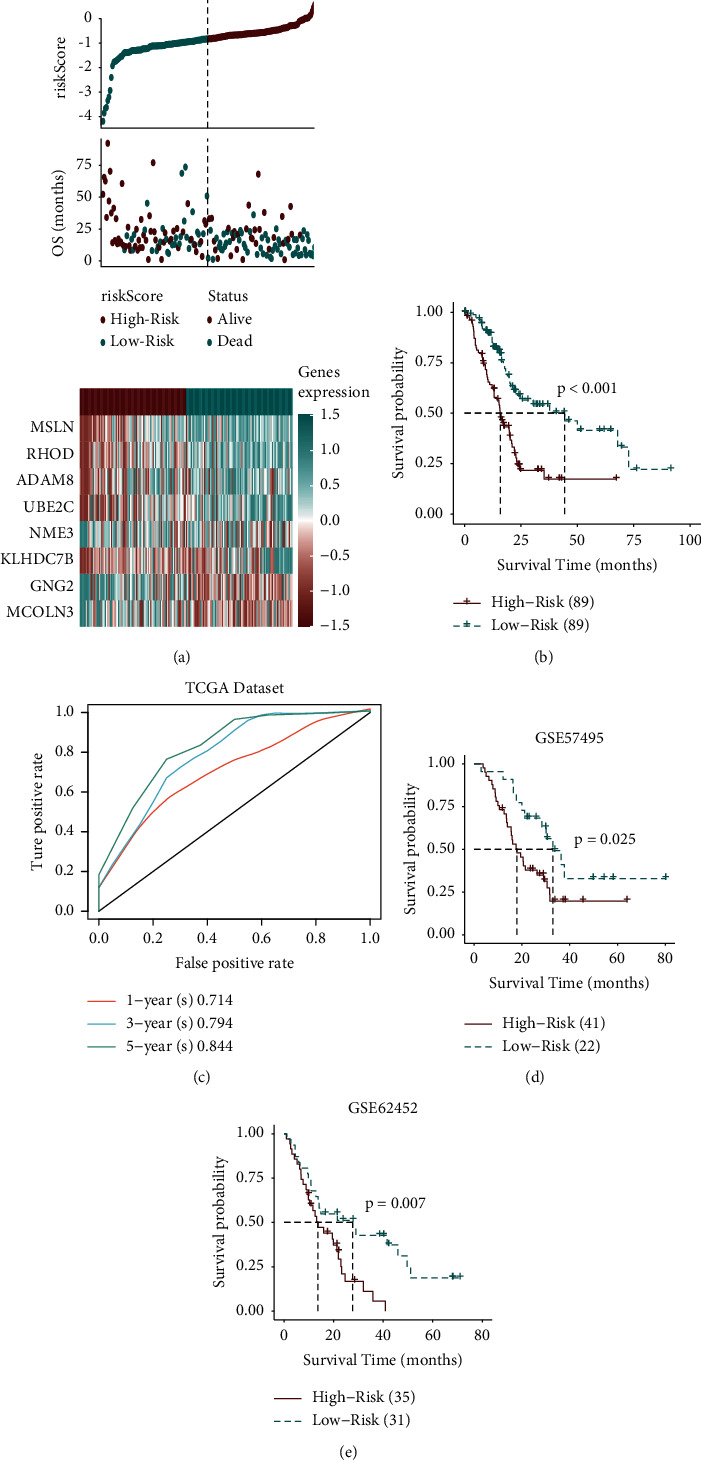
Analysis of TNF family genes and the prognosis of PAAD. (a) A composite graph consisting of risk score, survival status, and gene expression. (b) TCGA survival analysis indicated that patients with a high-risk score had a poor prognosis. (c) The time-dependent ROC diagram showed the AUC values of the model in 1 year, 3 years, and 5 years. (d) Survival analysis based on the GSE5749 dataset. (e) Survival analysis based on the GSE62452 dataset.

A total of 566 genes were identified based on differential analysis of the two TNF family genes (Figure 2(a)), 377 genes were obtained after single-factor screening and 34 genes were obtained by the SVM method (Figure 2(b)). Then, the model established by SVM back-deduced the clustering type, and the ROC reached 0.927 (Figure 2(c)). After Lasso analysis of the screened genes, a risk score model containing 8 genes (RHOD, UBE2C, KLHDC7B, MSLN, ADAM8, NME3, GNG2, and MCOLN3) was obtained (Figures 2(d) and 2(e)). The expression levels of these 8 genes and the corresponding regression coefficients were used to construct the risk formula: risk score = −0.0787^*∗*^MCOLN3 + 0.1609^*∗*^KLHDC7B + −0.1329^*∗*^GNG2 + 0.1735^*∗*^RHOD + −0.4003^*∗*^NME3 + 0.0913^*∗*^UBE2C + 0.0983^*∗*^MSLN + −0.0752^*∗*^ADAM8. Among the 8 genes, rhoD, UBE2C, KLHDC7B, MSLN, and ADAM8 with HR higher than 1 were regarded as high-risk factors, while the other 3 (NME3, GNG2, and McLN3) with HR less than 1 were regarded as protective factors (Figure 2(f)). According to the risk score formula, the optimal cutoff value = −1. Patients were divided into the high-risk group (risk score ≥ -1) and the low-risk group (risk score < −1) to assess the robustness of these 8 genes in predicting the OS in clinical practice of PAAD patients (Figure 3(a)). The low-risk group had better OS status than the high-risk group (Figure 3(b)). As can be seen from the TCGA survival analysis in Figure 3(c), the prognosis of PAAD patients in the high-risk group was worse than that in the low-risk group. The time-dependent ROC diagram showed that the AUC value of the model was relatively high in 1 year (AUC = 0.714), 3 years (AUC = 0.794), and 5 years (AUC = 0844), suggesting that the model is more accurate and has strong applicability (Figure 3(c)). Survival analysis of the GSE57495 and GSE62452 datasets showed that patients in the high-risk group had a worse prognosis (Figures 3(d) and 3(e)). Analysis in combination with the risk model and clinical factors (including age, gender, grade, stage, T, N, and M) indicated that the risk score was an independent prognostic risk factor (*P* < 0.01, HR = 4.286, 95% CI = 2.516–7.301) (Figures S1A and S1B).

### 3.2. Functional Analysis of the Prognostic Model

**Figure 4:**
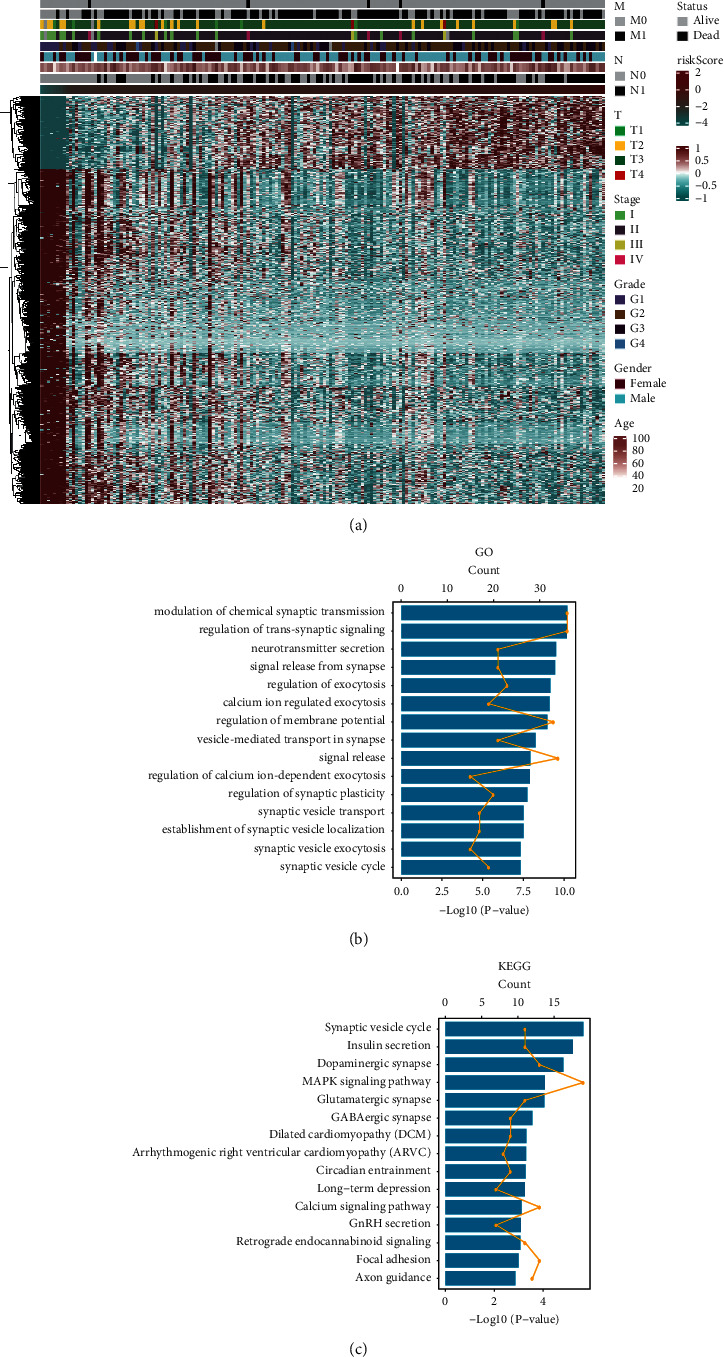
Functional analysis of prognostic models. (a) Display of genes associated with the risk score. (b) GO enrichment analysis and (c) KEGG enrichment analysis of related gene functions.

To understand the function of the obtained risk score-related genes, we first conducted correlation analysis of risk score-related genes and clinical characteristics, as shown in Figure 4(a). To further understand the potential functions of these genes, GO and KEGG enrichment analyses were performed, respectively. As shown in Figure 4(b), GO analysis revealed that these genes mainly participate in the modulation of chemical synaptic transmission, regulation of trans-synaptic signaling, neurotransmitter secretion, signal release from synapse, regulation of exocytosis, calcium ion-regulated exocytosis, regulation of membrane potential, vesicle-mediated transport in synapse, signal release, regulation of calcium ion-dependent exocytosis, regulation of synaptic plasticity, synaptic vesicle transport, establishment of synaptic vesicle localization, synaptic vesicle exocytosis, and synaptic vesicle cycle. KEGG analysis presented that these genes were primarily connected with synaptic vesicle cycle, insulin secretion, and dopaminergic synapse. These results manifest that the functions of these genes are mainly embodied in the regulation of information transmission between cells and the transfer of nanoparticles.

In addition, we conducted GSEA to comprehensively define the features of risk score. The hallmark gene set enrichment analysis showed that risk score-related genes were enriched in MYC targets V2 and TGF-*β* signaling (Figure S5A). GO enrichment analysis found that the genes were enriched in pore complex assembly and cysteine-type endopeptidase activity involved in the apoptotic process (Figure S5B). KEGG enrichment analysis revealed that the genes were concentrated in pathways involving pentose phosphate pathway (Figure S5C).

### 3.3. Immune Infiltration and Inflammation Analysis

**Figure 5:**
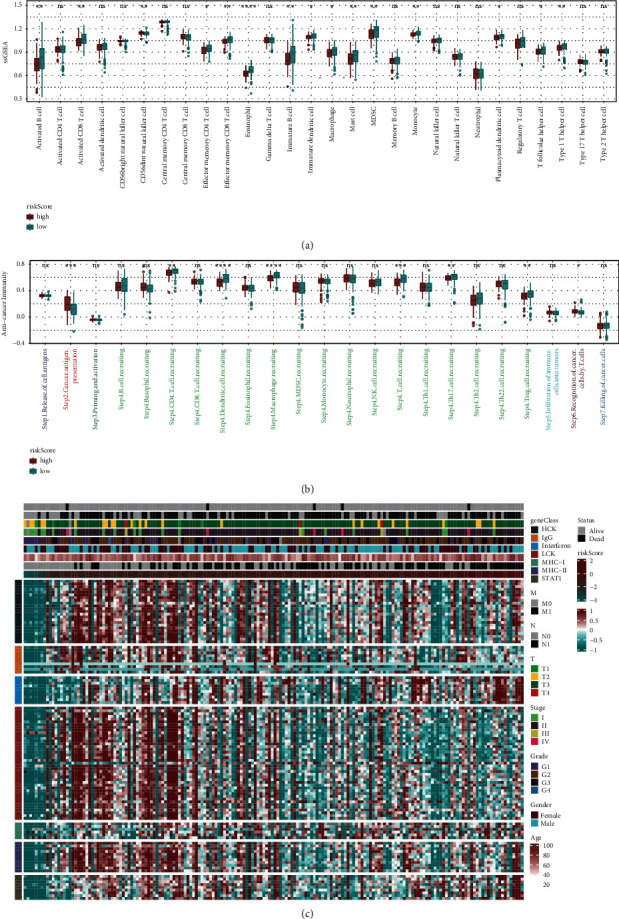
Immune and inflammatory analysis. (a) Differences in the expression of immune cells between the high-risk score group and low-risk score group. (b) Differences in the expression of immune-cycle activity scores between the high-risk score group and low-risk score group. (c) Changes in gene expression of different inflammatory marker gene sets.

It is well known that immune cell infiltration is closely related to inflammation. To this end, the ssGSEA method was used for immunoenrichment analysis of 28 kinds of immune cells. As shown in Figure 5(a), the expression of 15 kinds of immune cells showed a notable difference between the high-risk group and low-risk group, including activated B cell, activated CD8 T cell, CD56dim natural killer cell, effector memory CD4 T cell, effector memory CD8 T cell, eosinophil, immature B cell, immature dendritic cell, macrophage, mast cell, MDSC, monocyte, plasmacytoid dendritic cell, T follicular helper cell, and type 1 T-helper cell. Then, the immune-cycle activity scores of the two groups were statistically analyzed. We noted that Step2 (cancer antigen presentation), Step4 (including CD4 T cell, dendritic cell, macrophage, T cell, TH17 cell, and Treg cell recruitment), and Step6 (recognition of cancer cell by T cell) displayed statistical differences in anticancer immunity between the two groups (Figure 5(b)). As the risk score changed, the expression of genes in different inflammatory marker gene sets also changed accordingly, as shown in Figure 5(c). Genome set variation analysis (GSVA) was used to analyze the results of these 7 gene classes: HCK, IgG, interferon, LCK, MHC-I, MHC-II, and STAT1. After analyzing the correlation between the risk score and inflammatory indicators, we noticed that the risk score was significantly positively correlated with HCK, interferon, MHC-I, and STAT1 (*P* < 0.05) (Figure S2).

### 3.4. Prediction of the Risk Score for Immunotherapy Response

We used the TIDE algorithm to verify the risk score of the anti-PD-1 immunotherapy cohorts IMvigor 210 and GSE78220. In the GSE78220 cohort, the risk score in the complete/partial response (CR/PR) group was low compared to the stable/progressive disease (SD/PD) group (*P* = 0.04, Figure S6A). The CR/PR group had a higher percentage of scores than in the SD/PD group in the IMvigor 210 cohort (Figures S6B and S6C). Besides, the GSE79668 cohort confirmed that the prognosis of the high-risk group was worse than that of the low-risk group (Figure S6D).

### 3.5. Immune Checkpoint Analysis

**Figure 6:**
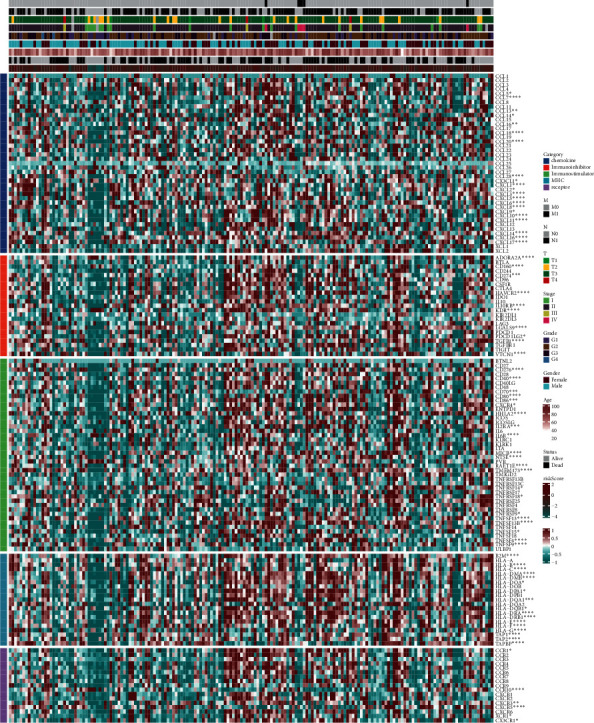
Expression of immune checkpoints as risk scores varied from low to high. ^*∗*^A correlation with the immune score.

We found that 21 immune checkpoints in chemokines were significantly correlated with the risk score, such as CCL5, CCL7, CCL13, CCL14, CCL16, CCL18, CCL20, and CCL28. 10 immune checkpoints such as CD160, CD274 and VTCN in Immunoinhibitor were associated with riskscore strongly. Immunostimulators included 21 immune checkpoints including CD276, CD40, CD70, CD80, and CD86, which were statistically correlated with the risk score. 17 immune checkpoints (including B2M, HLA-B, -C, -DMA, -DMB, and -DOA) in MHC and 6 immune checkpoints (CCR1, CCR10, CXCR3, CXCR5, XCR1, and CX3CR1) in receptors were significantly correlated with the risk score (Figure 6(a)). The expression of immune checkpoints changed as the risk score changed from low to high.

We also analyzed the correlation between risk scores and classical immune checkpoint molecules based on antigen present, cell adhesion, coinhibitor, costimulator, ligand, receptor, and other classifications. We found that the risk score was positively correlated with MICA, ICAM1, CD276, CD80, and TNFSF9 (*P* < 0.05, Figures S7A–E), and negatively correlated with ADORA2A and AEG1 (*P* < 0.05, Figures S7F–G).

## 4. Discussion

Through bioinformatics analysis, we noted that the TNF family genes were significantly associated with the clinical characteristics of PAAD patients. Our prognostic model based on TNF family genes has been proved clinically adaptable in predicting OS in PAAD patients. The risk score is an independent prognostic risk factor. In the PAAD prognostic model, the expression of immune cells, immune-cycle activity, and inflammatory markers was closely correlated with risk assessment. There was also obvious relativity between risk scores and immune checkpoints.

The tumor microenvironment of PAAD is highly immunosuppressive [18]. The complex tumor microenvironment has become one of the challenges that impedes PAAD treatment and leads to immune escape of pancreatic malignant cells [19]. TNF is not only a pleiotropic cytokine that triggers NF-*κ*B activation or RIPK1 kinase-dependent cell death but also a major mediator in inflammation [20]. As a type II transmembrane protein, TNF-*α* binds to tumor necrosis factor receptor 1 (TNF-R1) and TNF-R2, which subsequently activates downstream signaling pathways [7, 21]. Interestingly, TNF plays a “double-edged sword” role in cancer, largely depending on the role of TNF-R1 and TNF-R2 [22, 23]. TNF acts as a cancer suppressor by binding to TNF-R1. TNF-R2, on the other hand, can transform the tumor-suppressive TNF into the tumor promoter [24]. TNF-*α* and TGF-*β*, members of the TNF family, also play an important role in regulating TME [25, 26]. Low levels of TNF-*α* could increase tumor growth by inducing recruitment of endothelial phenotypes of monocytes to the tumor site [27]. Some researchers have proposed that local enhancement of endogenous TNF-*α* activity can accelerate the death of tumor cells without the associated systemic toxicity [28]. TNF/TNFR superfamily proteins are the major regulatory factors of T cells, among which Fas, TNF-R1, and TRAILR play an important role in promoting apoptosis and inhibiting T-cell activity [29]. Besides, TNFSF10 polymorphism has been identified as a possible prognostic factor for survival in patients undergoing surgery for invasive breast cancer [30].These encouraging studies hint at the potential of TNF family members in their efforts to diagnose and predict cancer. In this study, we clustered PAAD into two classes according to TNF family genes, acquired nineteen TNF family genes that were significantly correlated with the clinical characteristics (M, N, T, stage, grade, gender, and age) of PAAD patients, and further screened and established a risk score model. This model has been shown to have certain clinical prognostic value in PAAD.

Stimulation or inhibition of TNF superfamily signaling pathways may influence tumor progression [31]. We compared our model with the model established by Rong et al. [32]. In terms of sample size, our study included more samples (*n* = 178). Moreover, based on the time-dependent ROC results, the AUC of our model at 1 year, 3 years, and 5 years are 0.714, 0.794, and 0.844, relatively (Figure 3(c)). The 1-year, 3-year, and 5-year AUCs in the model established previously were 0.707, 0.75, and 0.795, respectively (Figure S4). This means that our model has better adaptability. We also noticed an interesting finding that the sensitivity of risk scores to predict 1-year, 3-year, and 5-year survival rates gradually increased. The risk factors included in our model, such as UBE2C and ADAM8, and their high expression levels are associated with poor clinical outcomes [33, 34]. Therefore, we speculate that the increased sensitivity of the risk score to survival may be due to the stronger expression of related genes with the development of PAAD.

Immune cells account for nearly 50% of the components of pancreatic ductal adenocarcinoma cells, but only a few are antitumor effector cells [35]. A novel study pointed out that monocytes may be an effective predictor of response to treatment in patients with glioma [36]. Our research also noticed that monocytes were significantly different between the high-risk group and low-risk group. Immunoinfiltration of macrophages has been used as a prognostic factor to assess the immune microenvironment of pancreatic ductal carcinoma [37]. This study found a significant correlation between the TNF family-related risk score model and the immune infiltration and inflammatory indicators. Immune checkpoint blockade (ICB) therapy is one of the most promising immunotherapies, especially in inhibiting metastasis. However, due to the immunosuppressive tumor microenvironment and extensive fibrotic matrix, immunotherapy is still greatly hindered in the treatment of pancreatic cancer [38]. For example, ICBs with programmed cell death protein-1 (PD-1)/programmed cell death ligand 1 (PD-L1) antibodies showed a sustained response rates in immunogenic tumors [39]. Patients with a large number of tumor-infiltrating lymphocytes in pancreatic ductal adenocarcinoma after ICB apparently have a better prognosis, while patients with mismatched repair defects have a better outcome, suggesting the possibility of a comprehensive immune enhancement that reverses the tumor microenvironment [40]. Through the analysis of different categories of immune checkpoints, we found that immune checkpoints changed as risk scores changed from low to high, and there was a strong correlation between the two. Zhang et al. proposed that the exploration of more valuable PD-L1 and CTLA-4 modulators to improve the efficacy of immunotherapy is currently an effective strategy to promote personalized cancer therapy [41].

In conclusion, the risk score model based on TNF family has good clinical value and adaptability in the prognosis of PAAD. Patients with high-risk PAAD tend to have a poorer prognosis, particularly with respect to immune infiltration and inflammation. In addition, we noted a strong correlation between risk scores and immune checkpoints. Our study may provide novel guidance for the diagnosis, prognosis, and treatment of PAAD.

## Data Availability

All data supporting this study are available from the corresponding author upon reasonable request.

## Supplementary Materials

Supplementary Materials Figure S1 A and B. The forest plot based on risk score and clinical factors (including age, gender, grade, stage, T, N, and M). Figure S2. The correlation between the risk score and inflammatory indicators. Figure S3. Univariate cox regression analysis of the prognostic value of TNF family members. Figure S4. The ROC curve measuring the sensitivity of the risk score in predicting a PAAD patient's 1-year, 3-year, and 5-year survival status, according to a splicing factor-based prognostic index. Figure S5. Gene set enrichment analysis of the risk score. (A) Hallmark gene set enrichment analysis. (B) GO enrichment analysis. (C) KEGG enrichment analysis. NES, normalized enrichment score. Figure S6. The anti-PD-1 immunotherapy cohort validated the predictive value of risk scores. (A) The risk score predicts immunotherapy response of GSE78220. (B) The risk score predicts immunotherapy response of IMvigor 210. (C) The proportions of CR/PR and SD/PD patients in low- and high-risk score groups. (D) The Kaplan–Meier curve of risk score in the GSE79668 cohort. Figure S7. Correlation between the risk score and classical immune checkpoint molecules. (A) Correlation between the risk score and MICA. (B) Correlation between the risk score and ICAM1. (C) Correlation between the risk score and CD276. (D) Correlation between the risk score and CD80. (E) Correlation between the risk score and TNFSF9. (F) Correlation between the risk score and ADORA2A. (G) Correlation between the risk score and AEG1.
