# Supplementary figures and images for: Molecular Analysis of Prognosis and Immune Pathways of Pancreatic Cancer Based on TNF Family Members
Source: J Oncol. 2021 Sep 30;2021:2676996. doi: 10.1155/2021/2676996 (PMC8497127; doi:10.1155/2021/2676996)

**A**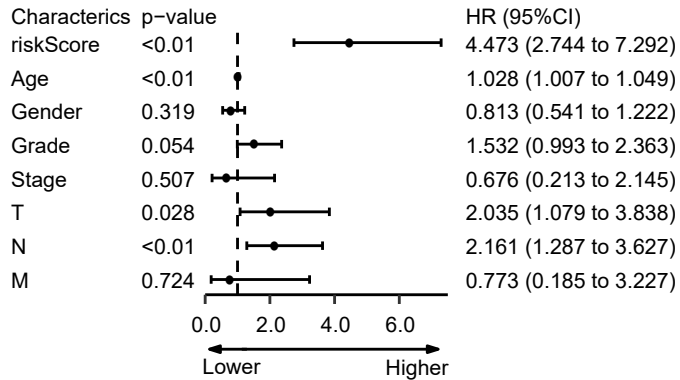**B**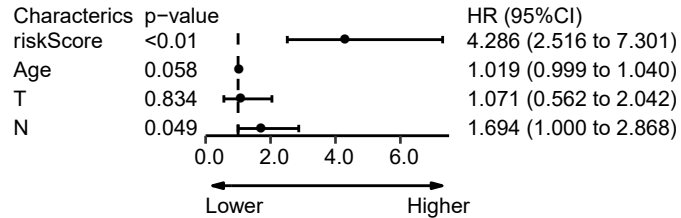

Supplement: Supplementary Materials — Figure S1 A and B. The forest plot based on risk score and clinical factors (including age, gender, grade, stage, T, N, and M). Figure S2. The correlation between the risk score and inflammatory indicators. Figure S3. Univariate cox regression analysis of the prognostic value of TNF family members. Figure S4. The ROC curve measuring the sensitivity of the risk score in predicting a PAAD patient's 1-year, 3-year, and 5-year survival status, according to a splicing factor-based prognostic index. Figure S5. Gene set enrichment analysis of the risk score. (A) Hallmark gene set enrichment analysis. (B) GO enrichment analysis. (C) KEGG enrichment analysis. NES, normalized enrichment score. Figure S6. The anti-PD-1 immunotherapy cohort validated the predictive value of risk scores. (A) The risk score predicts immunotherapy response of GSE78220. (B) The risk score predicts immunotherapy response of IMvigor 210. (C) The proportions of CR/PR and SD/PD patients in low- and high-risk score groups. (D) The Kaplan–Meier curve of risk score in the GSE79668 cohort. Figure S7. Correlation between the risk score and classical immune checkpoint molecules. (A) Correlation between the risk score and MICA. (B) Correlation between the risk score and ICAM1. (C) Correlation between the risk score and CD276. (D) Correlation between the risk score and CD80. (E) Correlation between the risk score and TNFSF9. (F) Correlation between the risk score and ADORA2A. (G) Correlation between the risk score and AEG1. [file 2676996.f1.zip › 2676996.f1/Figure S1.pdf]

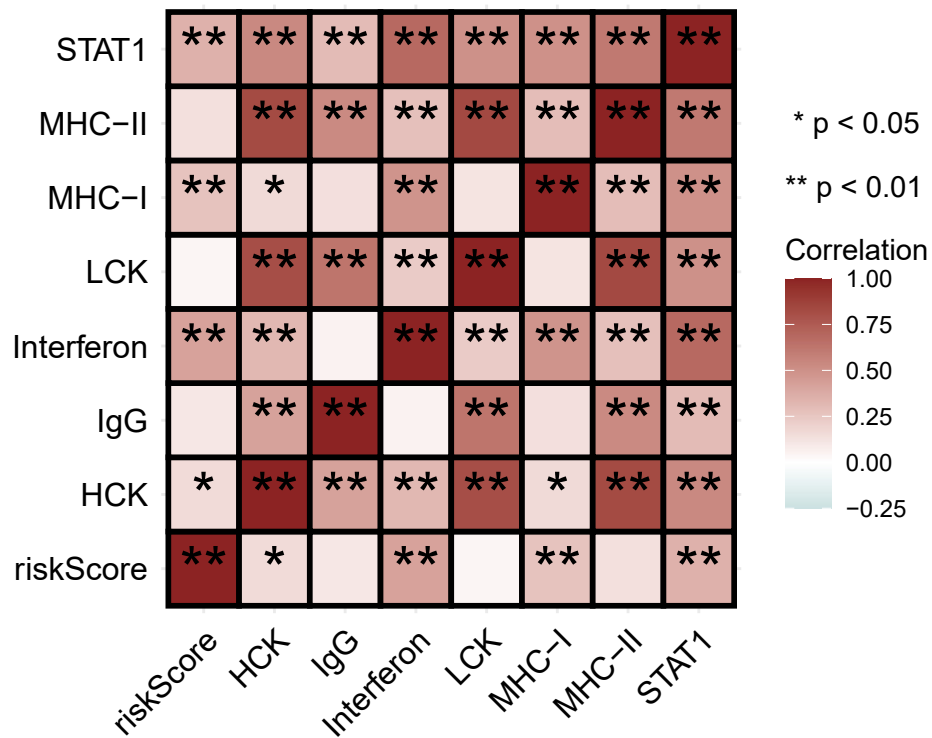

Supplement: Supplementary Materials — Figure S1 A and B. The forest plot based on risk score and clinical factors (including age, gender, grade, stage, T, N, and M). Figure S2. The correlation between the risk score and inflammatory indicators. Figure S3. Univariate cox regression analysis of the prognostic value of TNF family members. Figure S4. The ROC curve measuring the sensitivity of the risk score in predicting a PAAD patient's 1-year, 3-year, and 5-year survival status, according to a splicing factor-based prognostic index. Figure S5. Gene set enrichment analysis of the risk score. (A) Hallmark gene set enrichment analysis. (B) GO enrichment analysis. (C) KEGG enrichment analysis. NES, normalized enrichment score. Figure S6. The anti-PD-1 immunotherapy cohort validated the predictive value of risk scores. (A) The risk score predicts immunotherapy response of GSE78220. (B) The risk score predicts immunotherapy response of IMvigor 210. (C) The proportions of CR/PR and SD/PD patients in low- and high-risk score groups. (D) The Kaplan–Meier curve of risk score in the GSE79668 cohort. Figure S7. Correlation between the risk score and classical immune checkpoint molecules. (A) Correlation between the risk score and MICA. (B) Correlation between the risk score and ICAM1. (C) Correlation between the risk score and CD276. (D) Correlation between the risk score and CD80. (E) Correlation between the risk score and TNFSF9. (F) Correlation between the risk score and ADORA2A. (G) Correlation between the risk score and AEG1. [file 2676996.f1.zip › 2676996.f1/Figure S2.pdf]

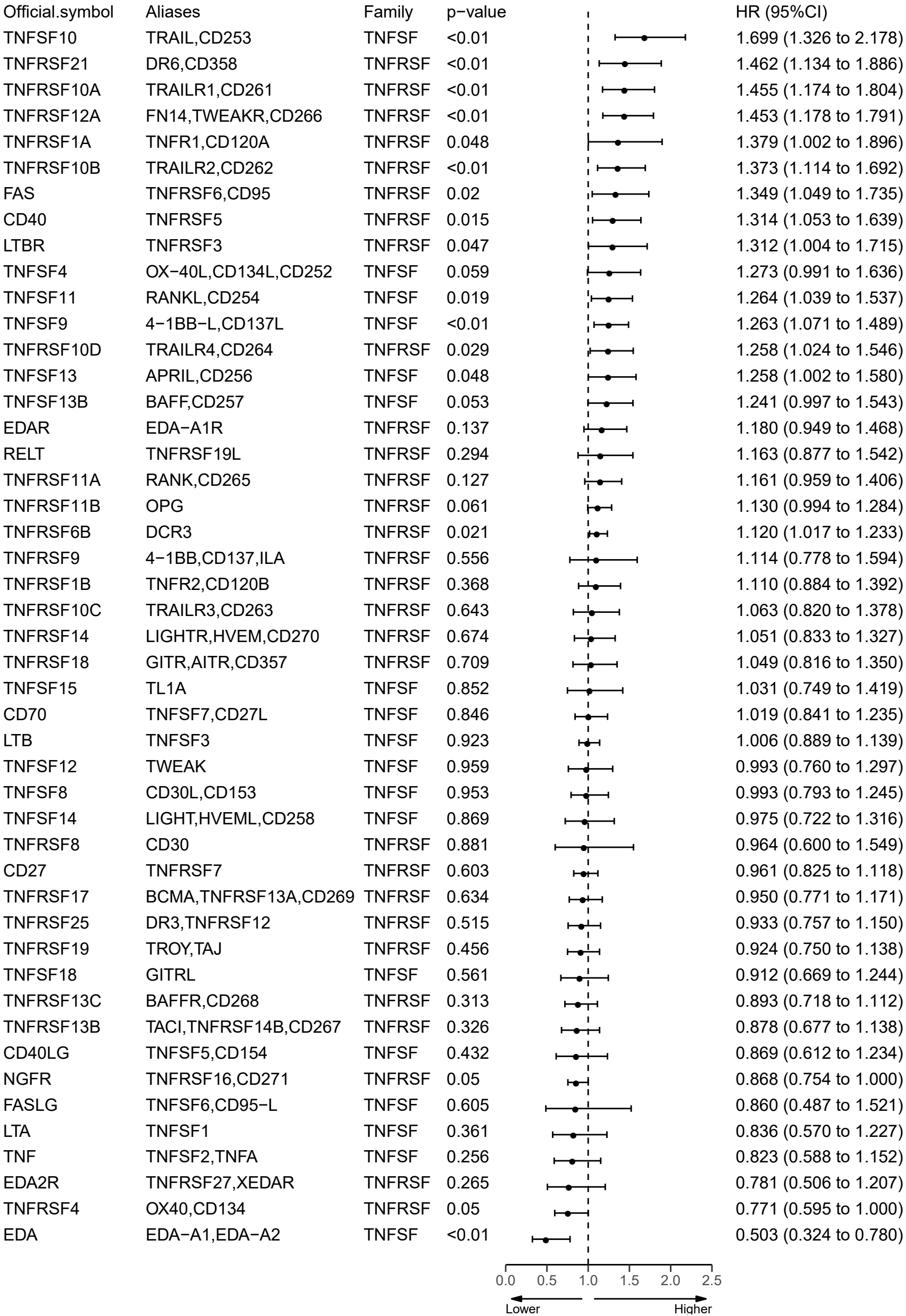

Supplement: Supplementary Materials — Figure S1 A and B. The forest plot based on risk score and clinical factors (including age, gender, grade, stage, T, N, and M). Figure S2. The correlation between the risk score and inflammatory indicators. Figure S3. Univariate cox regression analysis of the prognostic value of TNF family members. Figure S4. The ROC curve measuring the sensitivity of the risk score in predicting a PAAD patient's 1-year, 3-year, and 5-year survival status, according to a splicing factor-based prognostic index. Figure S5. Gene set enrichment analysis of the risk score. (A) Hallmark gene set enrichment analysis. (B) GO enrichment analysis. (C) KEGG enrichment analysis. NES, normalized enrichment score. Figure S6. The anti-PD-1 immunotherapy cohort validated the predictive value of risk scores. (A) The risk score predicts immunotherapy response of GSE78220. (B) The risk score predicts immunotherapy response of IMvigor 210. (C) The proportions of CR/PR and SD/PD patients in low- and high-risk score groups. (D) The Kaplan–Meier curve of risk score in the GSE79668 cohort. Figure S7. Correlation between the risk score and classical immune checkpoint molecules. (A) Correlation between the risk score and MICA. (B) Correlation between the risk score and ICAM1. (C) Correlation between the risk score and CD276. (D) Correlation between the risk score and CD80. (E) Correlation between the risk score and TNFSF9. (F) Correlation between the risk score and ADORA2A. (G) Correlation between the risk score and AEG1. [file 2676996.f1.zip › 2676996.f1/Figure S3.pdf]

# TCGA Dataset

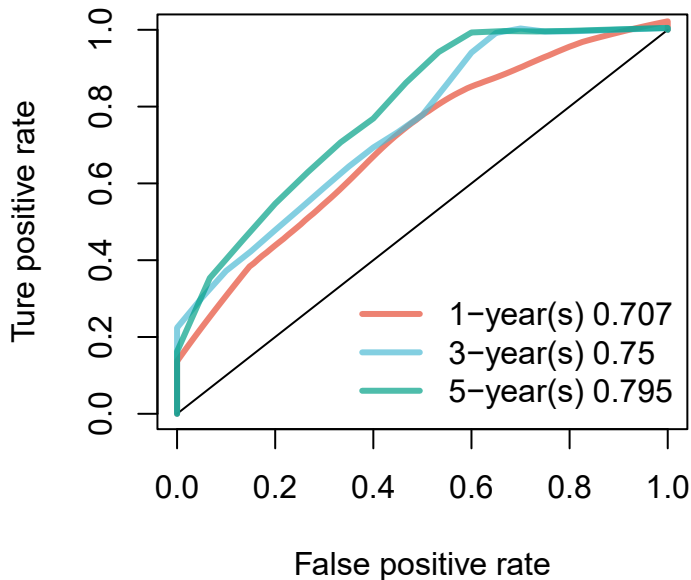

Supplement: Supplementary Materials — Figure S1 A and B. The forest plot based on risk score and clinical factors (including age, gender, grade, stage, T, N, and M). Figure S2. The correlation between the risk score and inflammatory indicators. Figure S3. Univariate cox regression analysis of the prognostic value of TNF family members. Figure S4. The ROC curve measuring the sensitivity of the risk score in predicting a PAAD patient's 1-year, 3-year, and 5-year survival status, according to a splicing factor-based prognostic index. Figure S5. Gene set enrichment analysis of the risk score. (A) Hallmark gene set enrichment analysis. (B) GO enrichment analysis. (C) KEGG enrichment analysis. NES, normalized enrichment score. Figure S6. The anti-PD-1 immunotherapy cohort validated the predictive value of risk scores. (A) The risk score predicts immunotherapy response of GSE78220. (B) The risk score predicts immunotherapy response of IMvigor 210. (C) The proportions of CR/PR and SD/PD patients in low- and high-risk score groups. (D) The Kaplan–Meier curve of risk score in the GSE79668 cohort. Figure S7. Correlation between the risk score and classical immune checkpoint molecules. (A) Correlation between the risk score and MICA. (B) Correlation between the risk score and ICAM1. (C) Correlation between the risk score and CD276. (D) Correlation between the risk score and CD80. (E) Correlation between the risk score and TNFSF9. (F) Correlation between the risk score and ADORA2A. (G) Correlation between the risk score and AEG1. [file 2676996.f1.zip › 2676996.f1/Figure S4.pdf]

A

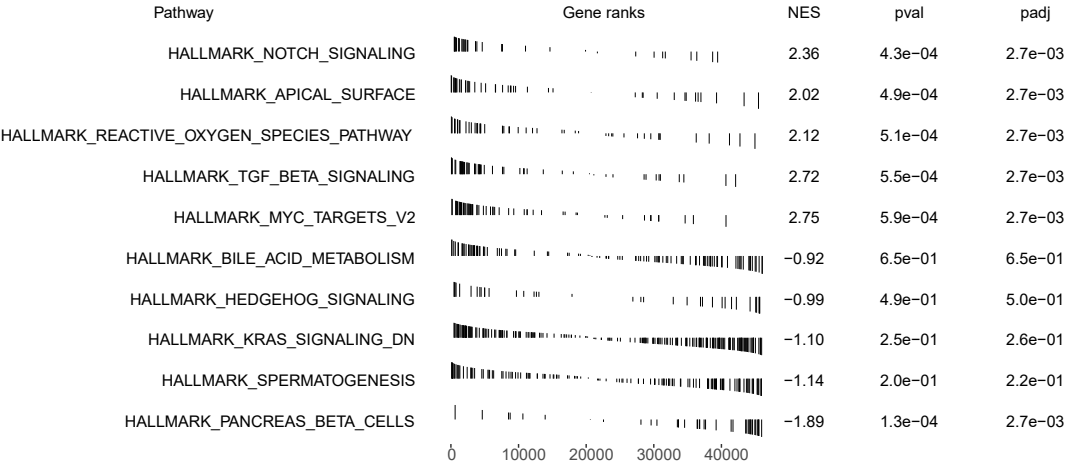

B

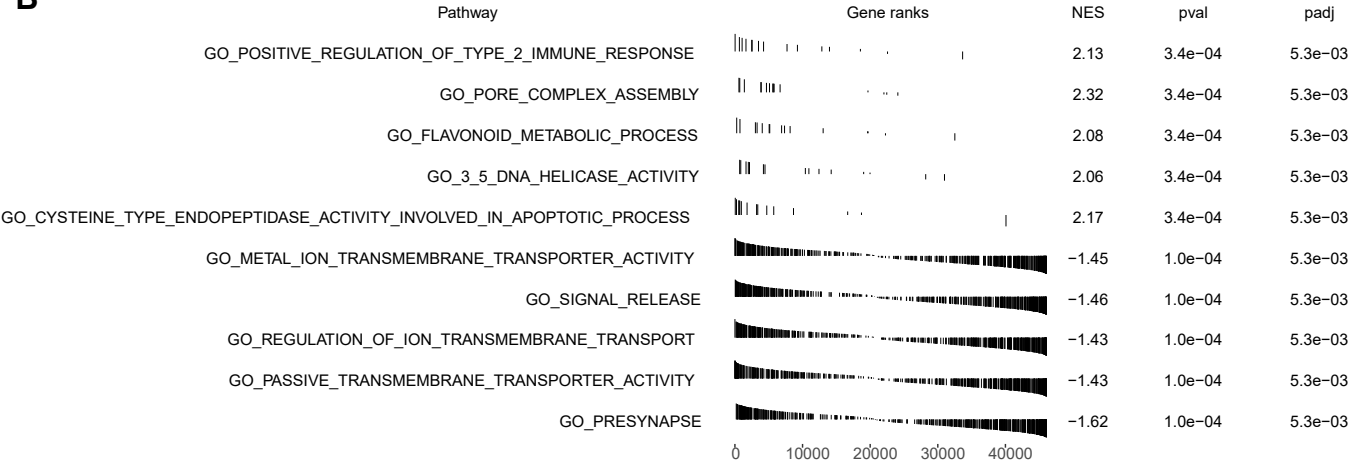

C

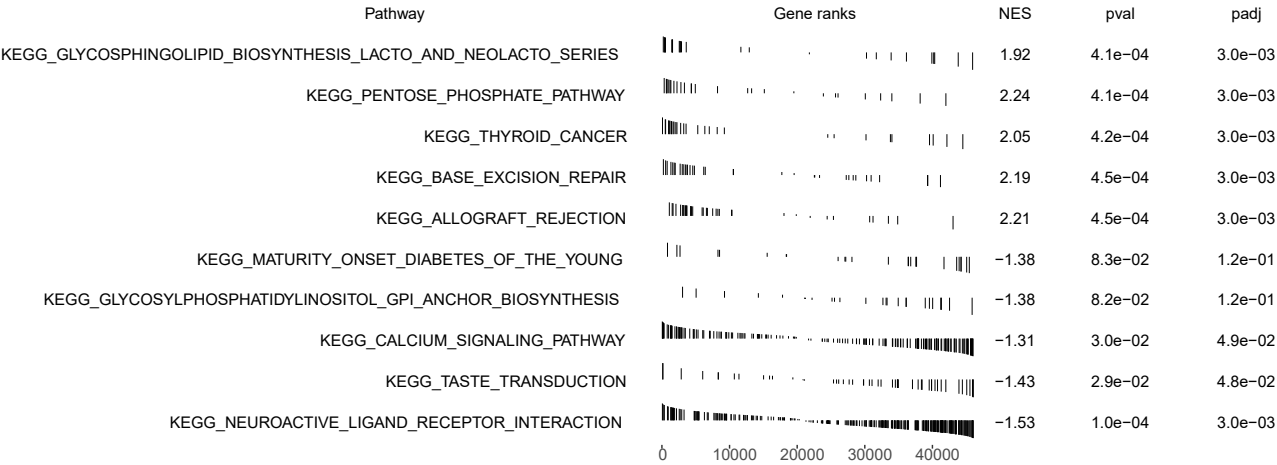

Supplement: Supplementary Materials — Figure S1 A and B. The forest plot based on risk score and clinical factors (including age, gender, grade, stage, T, N, and M). Figure S2. The correlation between the risk score and inflammatory indicators. Figure S3. Univariate cox regression analysis of the prognostic value of TNF family members. Figure S4. The ROC curve measuring the sensitivity of the risk score in predicting a PAAD patient's 1-year, 3-year, and 5-year survival status, according to a splicing factor-based prognostic index. Figure S5. Gene set enrichment analysis of the risk score. (A) Hallmark gene set enrichment analysis. (B) GO enrichment analysis. (C) KEGG enrichment analysis. NES, normalized enrichment score. Figure S6. The anti-PD-1 immunotherapy cohort validated the predictive value of risk scores. (A) The risk score predicts immunotherapy response of GSE78220. (B) The risk score predicts immunotherapy response of IMvigor 210. (C) The proportions of CR/PR and SD/PD patients in low- and high-risk score groups. (D) The Kaplan–Meier curve of risk score in the GSE79668 cohort. Figure S7. Correlation between the risk score and classical immune checkpoint molecules. (A) Correlation between the risk score and MICA. (B) Correlation between the risk score and ICAM1. (C) Correlation between the risk score and CD276. (D) Correlation between the risk score and CD80. (E) Correlation between the risk score and TNFSF9. (F) Correlation between the risk score and ADORA2A. (G) Correlation between the risk score and AEG1. [file 2676996.f1.zip › 2676996.f1/Figure S5.pdf]

**A**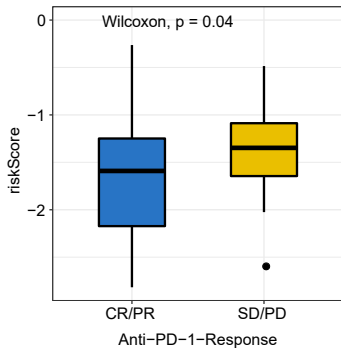**B**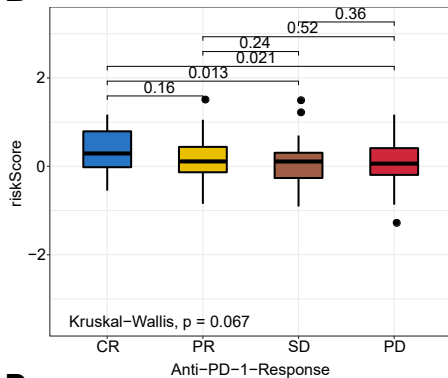**C**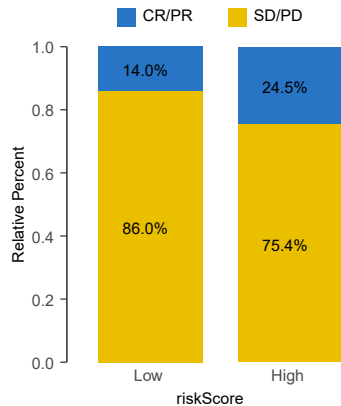**D**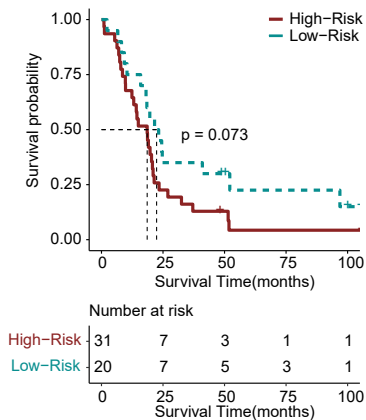

Supplement: Supplementary Materials — Figure S1 A and B. The forest plot based on risk score and clinical factors (including age, gender, grade, stage, T, N, and M). Figure S2. The correlation between the risk score and inflammatory indicators. Figure S3. Univariate cox regression analysis of the prognostic value of TNF family members. Figure S4. The ROC curve measuring the sensitivity of the risk score in predicting a PAAD patient's 1-year, 3-year, and 5-year survival status, according to a splicing factor-based prognostic index. Figure S5. Gene set enrichment analysis of the risk score. (A) Hallmark gene set enrichment analysis. (B) GO enrichment analysis. (C) KEGG enrichment analysis. NES, normalized enrichment score. Figure S6. The anti-PD-1 immunotherapy cohort validated the predictive value of risk scores. (A) The risk score predicts immunotherapy response of GSE78220. (B) The risk score predicts immunotherapy response of IMvigor 210. (C) The proportions of CR/PR and SD/PD patients in low- and high-risk score groups. (D) The Kaplan–Meier curve of risk score in the GSE79668 cohort. Figure S7. Correlation between the risk score and classical immune checkpoint molecules. (A) Correlation between the risk score and MICA. (B) Correlation between the risk score and ICAM1. (C) Correlation between the risk score and CD276. (D) Correlation between the risk score and CD80. (E) Correlation between the risk score and TNFSF9. (F) Correlation between the risk score and ADORA2A. (G) Correlation between the risk score and AEG1. [file 2676996.f1.zip › 2676996.f1/Figure S6.pdf]

**A**

Antigen present

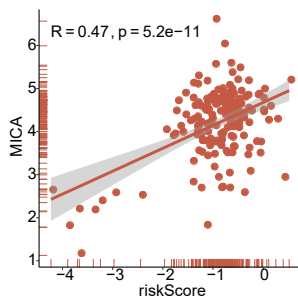**B**

Cell adhesion

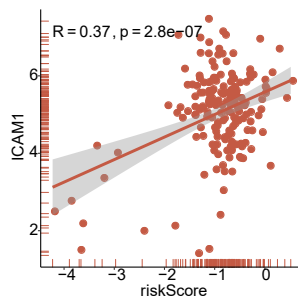**C**

Co-inhibitor

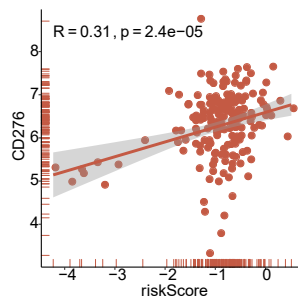**D**

Co-stimulator

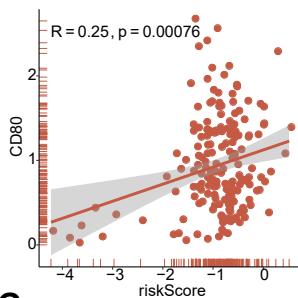**E**

Ligand

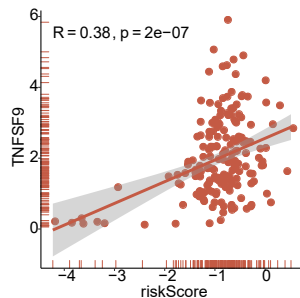**F**

Receptor

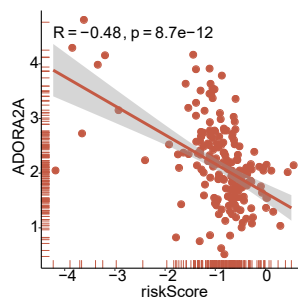**G**

Other

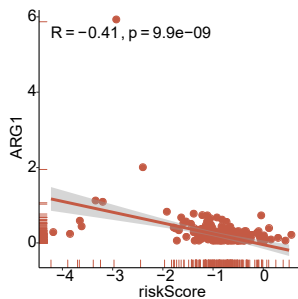

Supplement: Supplementary Materials — Figure S1 A and B. The forest plot based on risk score and clinical factors (including age, gender, grade, stage, T, N, and M). Figure S2. The correlation between the risk score and inflammatory indicators. Figure S3. Univariate cox regression analysis of the prognostic value of TNF family members. Figure S4. The ROC curve measuring the sensitivity of the risk score in predicting a PAAD patient's 1-year, 3-year, and 5-year survival status, according to a splicing factor-based prognostic index. Figure S5. Gene set enrichment analysis of the risk score. (A) Hallmark gene set enrichment analysis. (B) GO enrichment analysis. (C) KEGG enrichment analysis. NES, normalized enrichment score. Figure S6. The anti-PD-1 immunotherapy cohort validated the predictive value of risk scores. (A) The risk score predicts immunotherapy response of GSE78220. (B) The risk score predicts immunotherapy response of IMvigor 210. (C) The proportions of CR/PR and SD/PD patients in low- and high-risk score groups. (D) The Kaplan–Meier curve of risk score in the GSE79668 cohort. Figure S7. Correlation between the risk score and classical immune checkpoint molecules. (A) Correlation between the risk score and MICA. (B) Correlation between the risk score and ICAM1. (C) Correlation between the risk score and CD276. (D) Correlation between the risk score and CD80. (E) Correlation between the risk score and TNFSF9. (F) Correlation between the risk score and ADORA2A. (G) Correlation between the risk score and AEG1. [file 2676996.f1.zip › 2676996.f1/Figure S7.pdf]
